# Natural disease history of mouse models for limb girdle muscular dystrophy types 2D and 2F

**DOI:** 10.1371/journal.pone.0182704

**Published:** 2017-08-10

**Authors:** S. Pasteuning-Vuhman, K. Putker, C. L. Tanganyika-de Winter, J. W. Boertje-van der Meulen, L. van Vliet, M. Overzier, J. J. Plomp, A. Aartsma-Rus, M. van Putten

**Affiliations:** 1 Department of Human Genetics Leiden University Medical Centre, Leiden, The Netherlands; 2 Department of Neurology Leiden University Medical Centre, Leiden, The Netherlands; University of Minnesota Medical Center, UNITED STATES

## Abstract

Limb-girdle muscular dystrophy types 2D and 2F (LGMD 2D and 2F) are autosomal recessive disorders caused by mutations in the alpha- and delta sarcoglycan genes, respectively, leading to severe muscle weakness and degeneration. The cause of the disease has been well characterized and a number of animal models are available for pre-clinical studies to test potential therapeutic interventions. To facilitate transition from drug discovery to clinical trials, standardized procedures and natural disease history data were collected for these mouse models. Implementing the TREAD-NMD standardized operating procedures, we here subjected LGMD2D (SGCA-null), LGMD2F (SGCD-null) and wild type (C57BL/6J) mice to five functional tests from the age of 4 to 32 weeks. To assess whether the functional test regime interfered with disease pathology, sedentary groups were taken along. Muscle physiology testing of tibialis anterior muscle was performed at the age of 34 weeks. Muscle histopathology and gene expression was analysed in skeletal muscles and heart.

Muscle histopathology and gene expression was analysed in skeletal muscles and heart. Mice successfully accomplished the functional tests, which did not interfere with disease pathology. Muscle function of SGCA- and SGCD-null mice was impaired and declined over time. Interestingly, female SGCD-null mice outperformed males in the two and four limb hanging tests, which proved the most suitable non-invasive tests to assess muscle function. Muscle physiology testing of tibialis anterior muscle revealed lower specific force and higher susceptibility to eccentric-induced damage in LGMD mice. Analyzing muscle histopathology and gene expression, we identified the diaphragm as the most affected muscle in LGMD strains. Cardiac fibrosis was found in SGCD-null mice, being more severe in males than in females. Our study offers a comprehensive natural history dataset which will be useful to design standardized tests and future pre-clinical studies in LGMD2D and 2F mice.

## Introduction

Muscular dystrophies are a group of inherited, heterogeneous muscle disorders characterized by progressive muscle weakness and degeneration. The pattern of muscle weakness varies both in distribution and severity and underlies the subdivision into several disease types: Duchenne and Becker, Emery-Dreifuss, distal, facioscapulohumeral, oculopharyngeal, and limb-girdle muscular dystrophies [1]. The limb-girdle muscular dystrophies (LGMDs) involve a slowly progressive weakening of proximal muscles of the hip and shoulder girdles. LGMD is the most heterogeneous collection of muscular dystrophies with over 30 LGMD subtypes identified according to their genetic defects with autosomal dominantly and recessively inherited LGMDs subgrouped as LGMD1 and LGMD2, respectively. Sarcoglycanopathies are one of the more common forms of autosomal recessive LGMD and comprise four subtypes: LGMD2C, -D, -E and -F. They are caused by mutations in the genes coding for the muscle-specific transmembrane sarcoglycan proteins α-, β-, γ-, and δ-sarcoglycan. These sarcoglycans form a crucial component of the dystrophin-glycoprotein complex (DGC). The DGC is a large complex of membrane-associated proteins that further consists of dystrophin, the dystroglycans (α and β), sarcospan, the syntrophins (α1, β1, β2, γ1- and γ2) and α-dystrobrevin [2]. This complex physically links the intracellular cytoskeleton to the extracellular matrix. The loss of this structural connection, for instance due to mutations in one of the sarcoglycans, makes muscle fibers more susceptible to damage during muscle contraction [2–5].

Although the causative gene mutations are well characterized, there is no therapy available for sarcoglycanopathies [6]. Still, animal models for α- and δ-sarcoglycanopathies (LGMD types 2D and 2F) that recapitulate many features of human disease have been developed and are suitable for pre-clinical research [7, 8]. Although there is a growing demand for pre-clinical testing, there is a lack of generally accepted pre-clinical outcome measures and lack of natural history data for these outcome measures. Therefore, an international effort was initiated by the TREAT-NMD network and the Wellstone Center with the aim to develop standardized procedures focusing on pre-clinical models for Duchenne muscular dystrophy and spinal muscular dystrophy. These standardized procedures are used worldwide and permit more direct comparison of pre-clinical drug testing between laboratories (http://www.treat-nmd.eu/research/preclinical/overview/). There is a lack of natural history data and clearly defined outcome measures for LGMD mouse models.

Therefore, in the present study, we characterized the natural disease history of LGMD2D and 2F (SGCA- and SGCD-null) mice [9, 10] using standardized operating procedures available from the TREAD-NMD network for the *mdx* mouse model, which has a comparable phenotype. The data presented are useful for setting up standardized pre-clinical therapeutic trials in LGMD mice.

## Materials and methods

### Animals

The SGCA-null (B6.129S6-Sgca^tm2Kcam^/J, α-deficient) mice [9] were kindly provided by Queensta Millet, University College London and the SGCD-null (B6.129-Sgcd ^tm1Mcn^/J, δ-deficient) mice [10] were obtained from Jackson Laboratory (Bar Harbor, ME, USA) and bred in the Experimental Animal Facility of the Leiden University Medical Center. All mice used in this study were on a C57BL/6J background and were compared to C57BL/6J wild type mice. Mice were kept in individually ventilated cages with 12 hours of light/dark cycles at 20.5°C and had *ad libitum* access to standard RM3 chow (SDS, Essex, UK) and water. All experiments were approved by and performed following the guidelines of the Animal Experiment Committee (DEC 13211) of the Leiden University Medical Center. Care was taken to limit the burden and distress for the animals as much as possible.

### Functional test regime

SGCA-null and SGCD-null mice (12 mice per genotype; 6 males and 6 females) were randomly assigned to either a functionally challenged or unchallenged group. Wild type mice (C57BL/6J) (12 mice; 6 males and 6 females) were assigned to the functionally challenged group only, as the functional test regime is not detrimental to wild type mice [11]. Functionally challenged mice were subjected to a functional test regime consisting of five functional tests performed on consecutive days, from the age of 4 to 34 weeks twice monthly. Body weight was recorded at the start of each functional test regime session. Functional tests aimed to assess muscle function, strength, coordination and condition. An overview of the experimental timelines is given in the S1 Fig. Standardized operating procedures from the TREAT-NMD network were implemented when possible [12]. All experiments were performed blinded to avoid biased results.

#### Four limb grip strength test

Peak force of the fore and hind limbs was measured using a grid attached to an isometric force transducer (Columbus Instruments, USA). Hereto, the mouse was suspended, handled by the tail, above the grid which it naturally grasped with its four paws. It was then pulled from the grid. The maximal force applied by the mouse when pulled from the grid was recorded by the force transducer. This test was repeated three times in a row after which the mouse was allowed a short rest. The mouse was suspended to five series of three pulls, each followed by a resting period. In this way the mouse was pulled in total 15 times. The three highest values out of the 15 values obtained were averaged and the maximum grip strength was calculated and normalized to body weight.

#### Rotarod

Motor coordination and balance were assessed with rotarod running. The mouse was placed on the rotarod (Ugo Basile, Italy) that accelerated from 5 to 45 rotations per minute within 15 seconds. The time it took mice to fall off the rod was recorded. The test session was completed when the mouse had run for 500 seconds without falling. Mice that had fallen off within 500 seconds were allowed two more tries. The longest running time was used for analysis.

#### Two limb hanging test

The maximum hanging time with two limbs was assessed using a previously described protocol [12]. The test ended after a hanging time of 600 seconds was achieved or otherwise after three sessions. The maximum time score was used for analysis.

#### Four limb hanging test

The maximum hanging time with four limbs was performed using a previously described protocol [12].The test ended after a hanging time of 600 seconds was achieved or otherwise after three sessions. The maximum hanging time was used for analysis.

#### Beam walk test

The mouse was placed on an elevated narrow beam (diameter 11mm) and had to walk across to a safe black box, while being filmed. Performance on the beam was quantified by the time it took to traverse the 80 cm long beam and the numbers of hind paw slips that occurred during beam walking. The mice were allowed three tries. The traverse times and numbers of paw slips were averaged.

### Respiratory function analysis

All mice underwent respiratory function analysis with whole-body plethysmography [13] (RM-80; Columbus Instruments, Columbus, OH, USA) at the ages of 15 and 34 weeks. After 30 seconds acclimatization, the respiration signal was recorded for 120 seconds. The signal was digitized using a Minidigi digitizer and AXOSCOPE 10 software (Axon Instruments/Molecular Devices, Sunnyvale, CA, USA) and analysed with the event detection feature of the Clampfit 10 program (Axon Instruments/Molecular Devices). This non-invasive monitoring system provided breathing function analysis measuring respiration rate and depth. The respiration signal amplitude was normalized to body weight.

### Creatine kinase level analysis

Monthly before performing functional tests, blood was taken via a small angled cut in the tail and collected in heparin coated microvettes (Sarstedt B.V. the Netherlands) and stored on ice. To separate plasma, the blood was centrifuged at 4°C for five minutes at 13000 RPM. Plasma creatine kinase (CK) levels were measured with Reflotron CK test strips in the Reflotron plus machine (Roche Diagnostics Ltd., UK).

### *In vivo* force measurements of tibialis anterior muscles

At 34 weeks of age, each mouse was anesthetized with 2% isoflurane and function of the right tibialis anterior muscle was assessed using previously described protocol [14]. In short, after mice were surgically prepared, muscles underwent a warm up protocol (five stimulations of 50 Hz spaced one minute apart). Thereafter, the maximum isometric tetanic force (Po) was determined from the plateau of the force—frequency relationship following a series of stimulations at 10, 30, 40, 50, 80, 100, 120, 150 and 180 Hz. The specific force (N/cm^2^) was determined by dividing Po by the muscle cross-sectional area. The overall cross-sectional area was calculated using the formula: muscle weight (g)/ [tibialis anterior fiber length (Lf; cm) × 1.06 (g/cm^3^)]. The specific isometric force was determined by dividing the absolute force at each stimulation frequency by the muscle’s cross sectional area. After five minutes, susceptibility to contraction-induced injury was measured. To this end, muscles were stimulated at 120 Hz for 500 ms before lengthening at 10% of L_o_ at a velocity of 0.5 Lo s^-1^. At the end of the stimulation session, the muscle was returned to L_o_ at a rate of −0.5 Lo s^-1^. The stimulation-stretch cycle was repeated every two minutes to a total of ten cycles. The maximum isometric force of the first contraction served as the 100% baseline. After the procedure, the muscles were isolated, weighed and prepared for histological and gene expression analyses.

### Muscle histology and morphology

Frozen muscle sections (8 μm thick) were cut with a cryotome and intermediate sections were collected for RNA isolation. Slides were fixed in ice-cold acetone for five minutes, stained with hematoxylin and eosin (Sigma-Aldrich, Zwijndrecht, the Netherlands) and mounted in Permount mounting medium (Histolab, Västra Frölunda, Sweden), according to conventional methods. To localize and quantitate lipid droplets, muscle sections were stained with Nile red (9-diethylamino-5H-benzo(alpha)phenoxazine-5-one) staining (Thermo fisher scientific, Breda, the Netherlands) [15]. Non-fixed muscle sections were air dried, washed with PBS/Tween 0.05%, incubated in 1 μmol Nile red solution for 15 minutes and rinsed three times in PBS/Tween 0.05%. Sections were mounted with mounting medium with DAPI (ProLong Antifade Reagents; Life Technologies, Bleiswijk, the Netherlands) and imaged with an AF6000 (Leica) and BZ-X700 fluorescent microscopes (Keyence, Osaka, Japan) at ten times magnification. The percentage of fat accumulation of the entire muscle was quantified with ImageJ by dividing the area occupied with fat droplets by the total tissue area. For the fiber size distribution analyses, muscle sections were stained with a laminin primary antibody (ab11575, dilution 1:200 Abcam, USA) and with a goat-anti-rabbit Alexa 594 secondary antibody (A11012, dilution 1:1000, Life Technologies). The distribution was assessed with SMASH (Matlab based analysis) as previously described [16]. Five microscopic views were analysed resulting in an average total number of 1500–3000 fibers per muscle measured and the number of fibers in a given Feret’s diameter class (10 μm/class) was determined. To detect regenerative fibers, diaphragm muscle sections were stained with an eMHC primary antibody (F1.652, sc-53091 dilution 1:20, Santa Cruz Biotechnology, Inc) overnight at 4°C and with a goat-anti-mouse Alexa 488 secondary antibody (A11001, dilution 1:1000, Life Technologies) and DAPI for 1.5 hours at room temperature. Images of the stained muscle sections were taken with a BZ-X700 X700 fluorescent microscope (Keyence) at ten times magnification. For fibrotic area analyses, muscle and heart sections were stained with collagen type I primary antibody (1310–01 Southern Biotech, dilution 1:100, Birmingham, USA) overnight at 4°C and with a donkey-anti-goat Alexa 488 secondary antibody (A11001, dilution 1:1000, Life Technologies) and DAPI for one hour at room temperature and mounted with mounting medium (ProLong Antifade Reagents; Life Technologies). Images of the stained muscle and heart sections were taken with a BZ-X700 X700 fluorescent microscope (Keyence) at ten times magnification and stitched with the Leica software LASFx. Using ImageJ analysis the fibrotic areas were measured. The percentage of fibrotic area was calculated by dividing the collagen type I positive area by the total tissue area.

### RNA isolation and, qPCR

Muscle sections were collected in 1.4 mm Zirconium Beads prefilled tubes (OPS Diagnostics, Lebanon, USA) and homogenized in TriPure isolation reagent (Roche Diagnostics, Basel, Switzerland) using a MagNA Lyser (Roche Diagnostics). Total RNA was isolated using the TriPure isolation method and the RNA was further cleaned up by applying a NucleoSpin RNA II kit (Macherey-Nagel, Düren, Germany) according to the manufacturer's instruction. cDNA was synthesized from 0.4–1 μg of RNA (depending on the muscle type but kept constant between comparisons) using random N6 primers (Thermo fisher scientific) and Bioscript enzyme (GCBiotech, Alphen aan den Rijn, the Netherlands) according to the manufacturer's instructions. Quantitative PCR was performed in triplicate per biological sample using a LightCycler 480 and ready-to-use SensiMix reagents (GCBiotech). The expression levels were analysed with the LinReg qPCR method and normalized to expression values of the housekeeping gene *Gapdh*. Primer sequences and detailed PCR conditions can be provided on request.

### Statistical analyses

Data were analysed by using Prism 4 (GraphPad Software, La Jolla, CA, USA) and SPSS 17.0.2 (IBM, Armonk, NY, USA). Values are presented as means ±SD or ±SEM. A linear regression model was applied to compare the maximum hanging times of the hanging tests and the maximum running times from the rotarod tests over age per mouse per genotype. Area under curve (AUC) values were calculated for grip strength, CK levels, traverse latency and number of hind limb slips from beam walk test over age per mouse per genotype and then compared between genotypes with a one-way ANOVA test. Respiration amplitudes and rates were compared between genotypes using mixed models. For physiological analyses of tibialis anterior muscles, AUCs were calculated for the specific force over frequency per genotype and the initial force drop over number of contraction per genotype and compared between genotypes with a one-way ANOVA test. To compare maximal hanging time between genders, AUCs were calculated over age per mouse per gender and compared between genders with a one-way ANOVA test. Results of histological and gene expression analyses were compared between genotypes using a two-way ANOVA test and corrected for multiple comparisons with Tukey’s multiple comparison test. Fiber size distribution was assessed using logistic regression with SPSS to demonstrate the switch toward smaller fiber sizes for SGCA- and SGCD-null mice. Statistical significance was set at *P < 0*.*05*. SPSS statistics syntax can be provided on request.

## Results

### Impaired muscle function and muscle integrity in LGMD strains

SGCA-, SGCD-null and wild type mice were subjected twice monthly to a functional test regime from 4 until 32 weeks of age. The functional test regime consisted of five different functional tests: four limb grip strength, rotarod running, two and four limb hanging tests and beam walk test.

**Fig 1:**
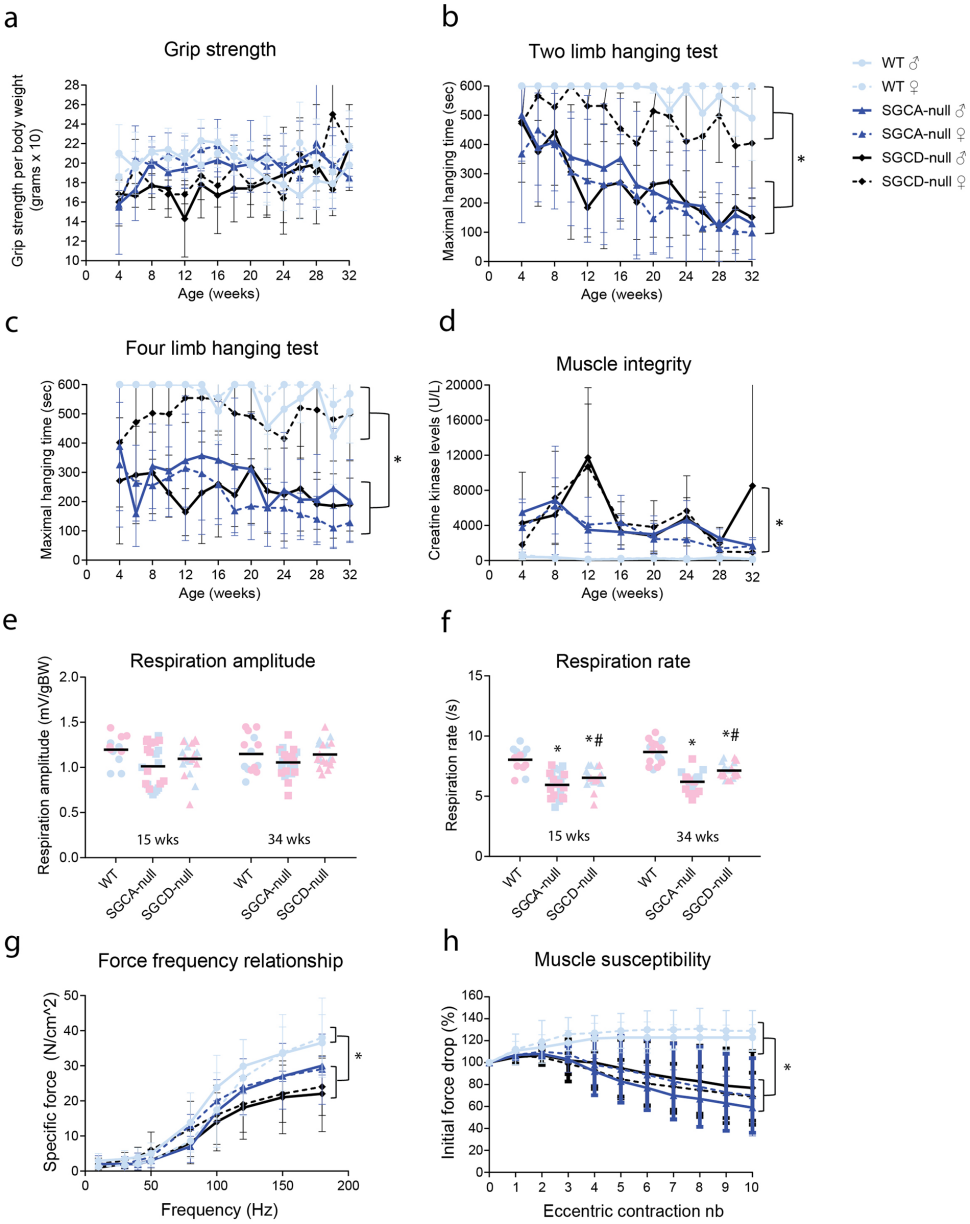
Impaired muscle function and integrity in SGCA- and SGCD-null mice. **(a)** Normalized four limb grip strength was significantly lower in SGCD-null mice than in wild type and SGCA-null mice **(b)** Maximum hanging time with two limbs was significantly shorter in SGCA- and SGCD-null mice when compared to wild type mice. SGCD-null mice outperformed SGCA-null mice. Maximum hanging time was significantly shorter in male SGCD-null mice than in female SGCD-null mice. **(c)** Maximum hanging time with four limbs was significantly shorter in SGCA- and SGCD-null mice when compared to wild type mice. SGCD-null mice outperformed SGCA-null mice. Maximum hanging time was significantly shorter in male SGCD-null mice than in female SGCD-null mice. **(d)** Creatine kinase levels were significantly elevated in both LGMD strains. **(e)** No significant differences were detected in respiration amplitude between the mouse models. **(f)** Respiration rate was significantly decreased in SGCA- and SGCD-null mice at 15 and 34 weeks of age compared to wild type mice. At 15 and 34 weeks of age, SGCA-null mice showed significantly lower respiration rate than SGCD-null mice. No differences were found between males (in blue) and females (in pink). **(g)** Force frequency relationship of the tibialis anterior muscle. Each data point represents the force measured at each frequency. Muscles of both SGCA- and SGCD-null mice showed a significantly lower specific force than those of wild type mice. SGCD-null muscles generated a significantly lower specific force than those of SGCA-null mice. **(h)** Relative changes in tetanic force during eleven cycles of eccentric contraction in tibialis anterior muscle. The tetanic tension developed during the first cycle was taken as 100%. The isometric force significantly dropped by 10–15% in SGCA- and SGCD-null mice, while it remained unchanged in wild type mice. For e and f * Indicates a significant difference from wild type (WT) controls. # Indicates a significant difference from SGCA-null mice. Error bars represent standard error of the mean (± SEM).

Since experimental groups consisted of six male and female mice, we could study differences in muscle performance not only between strains but also between genders. In the four limb grip strength test that served to assess muscle strength and function, the normalized grip strength did not differ either between strains or genders. However, combining male and female data resulted in a significant decrease in the normalized grip strength in SGCD-null mice when compared to SGCA-null and wild type mice over time (Fig 1a and S2a Fig). In the rotarod test, which examines coordination and endurance, both SGCA- and SGCD-null mice had a shorter running time than had wild type mice, but this did not reach significance due to high inter- individual variation in wild type mice (S2b Fig). Muscle strength and fatigability were tested using two and four limb hanging tests. Interestingly, female SGCD-null mice significantly outperformed male mice in two and four limb hanging tests, while no difference between genders was observed for SGCA-null or wild type mice (Fig 1b and 1c and S2c and S2d Fig). Moreover, SGCA- and male SGCD-null mice hung for a significantly shorter time period than did wild type mice in both tests (Fig 1b and 1c and S2c and S2d Fig). In contrast to the four limb grip strength data, combined data revealed that SGCA-null mice performed significantly worse in the hanging tests than did SGCD-null mice (S2c and S2d Fig), which can be explained by the better performance of SGCD females. In the two limb hanging test, the performance of both LGMD strains notably deteriorated over time (Fig 1b and S2c Fig). In addition, the performance of SGCA-null mice significantly declined over time in four limb hanging test, whereas there was no significant decline in SGCD-null mice (Fig 1c and S2d Fig). Muscle coordination and the ability to balance was assessed with the beam walk test (S2e and S2f Fig). The traverse times and numbers of paw slips were lower at baseline and increased over time in LGMD mice compared to wild type mice. Due to high inter-individual variation, differences did not reach significance.

Body weight and plasma CK levels were assessed longitudinally. No differences were found in body weights between LGMD and wild type mice (S2g Fig). SGCA- and SGCD-null mice had significantly higher plasma CK levels than wild type mice (<500 U/L). CK levels neither differed between the LGMD strains nor between female and male mice (Fig 1d).

As diaphragm muscle can be severely affected in dystrophic mouse models, we measured respiratory function with whole-body plethysmography in mice aged 15 and 34 weeks. While the respiration amplitude was similar across wild type, SGCA- and SGCD-null mice (Fig 1e), both SGCA- and SGCD-null mice showed significant lower respiration rates than did wild type mice at 15 and 34 weeks of age (Fig 1f). Similar to the hanging tests data, the respiration rate was significantly lower in SGCA- than in SGCD-null mice. However, the respiration amplitude and rate did not differ between genders.

We performed tibialis anterior physiology analysis with a focus on force-frequency relationship and resistance to eccentric contraction at 34 weeks of age. The force-frequency curve of fast-twitch tibialis anterior muscles stayed flat and rose rapidly from 50 Hz onward. The tibialis anterior muscles of SGCA- and SGCD-null mice generated a significantly lower specific force than those of wild type mice over a wide range of stimulation intensities (10–180 Hz) (Fig 1g). Unlike with the hanging tests, the specific force was comparable between males and females from the same strain. Furthermore, SGCD-null muscles generated a significantly lower specific force than SGCA-null muscles. We also measured the tibialis anterior muscle’s response to eccentric contraction, which can induce injury in dystrophic muscle fibers [17]. Tibialis anterior muscles were repetitively stimulated at 120 Hz and stretched to 110% of their resting length. While the isometric force significantly dropped by 10–15% in SGCA- and SGCD-null mice, it remained unchanged in wild type mice (Fig 1h). Similar to the specific force data, the isometric force measurements did not differ between genders from the same strain.

To summarize, mice successfully accomplished the functional test regime suggesting that it is suitable to use for both LGMD strains. Muscle function of SGCA- and SGCD-null mice was impaired and declined over time. In both hanging tests, marked differences in performance were detected in LGMD relative to wild type mice, while no differences were found in the rotarod and beam walk tests due to large inter-individual variation. Female SGCD-null mice outperformed males in the two and four limb hanging tests, while this was not the case for SGCA-null and wild type mice.

### Histological examination and gene expression analyses

#### Shift towards smaller fibers in LGMD muscles

**Fig 2:**
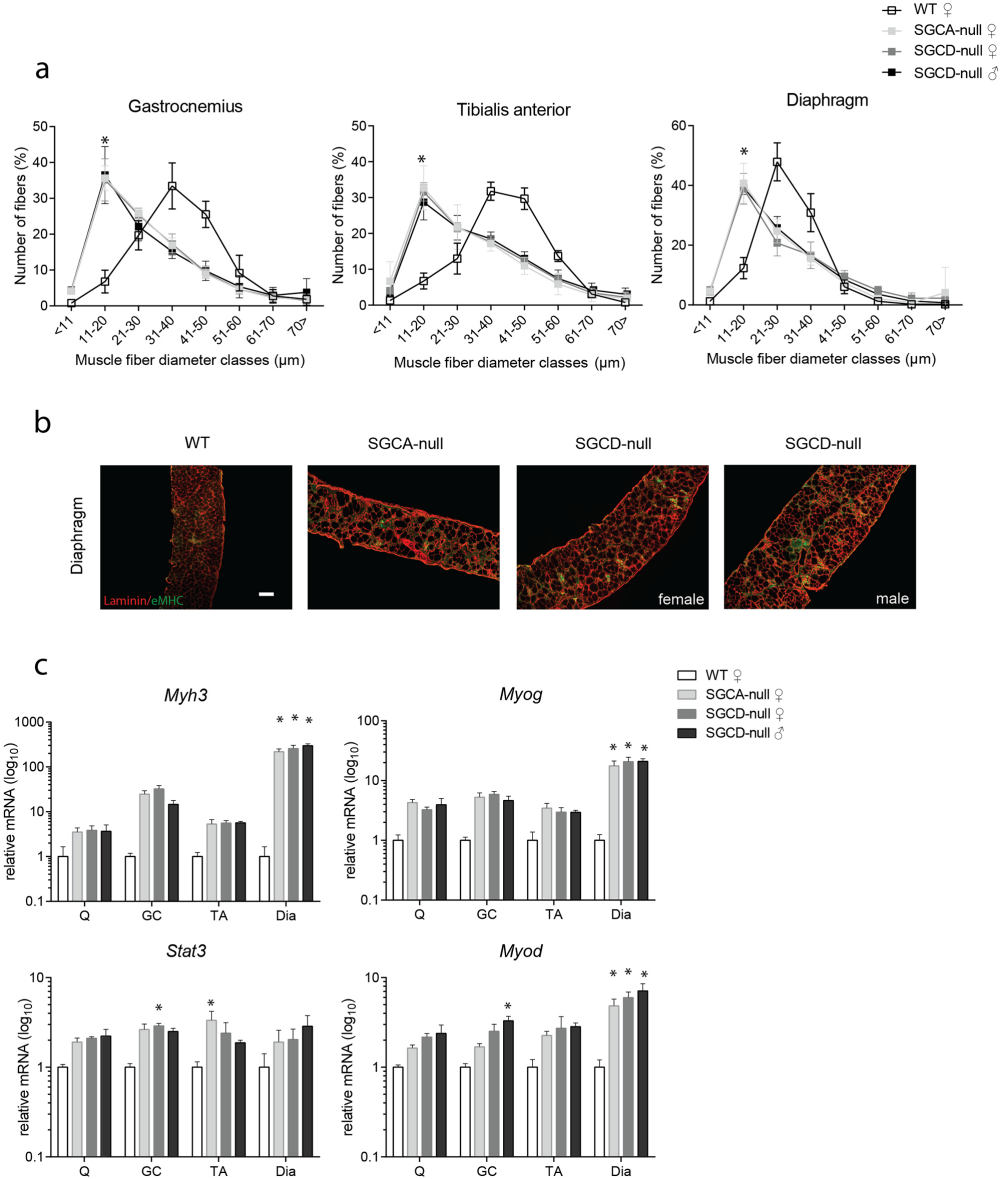
Smaller fiber sizes and increased muscle regeneration in SGCA- and SGCD-null mice. **(a)** Fiber size distribution of skeletal muscles of 34-week-old SGCA-null, SGCD-null and wild type mice. Values represent relative number of fibers in a given diameter class (10 μm/class). The fiber size distribution was shifted to smaller fibers in SGCA- and SGCD-null mice when compared to wild type mice in gastrocnemius, tibialis anterior and diaphragm muscles. Fiber size distribution did not differ between male and female SGCD-null mice. **(b)** Immunofluorescent images of diaphragm muscles stained with embryonic myosin heavy chain (eMHC) (regenerative marker, green) and laminin (extracellular matrix of muscle fibres, red) Scale bar: 100 μm. **(c)** Increase in myogenic gene expression (*Myh3*, *Myog*, *Stat3* and *Myod*) measured by qPCR and normalized to *Gapdh* in skeletal muscles of 34-week-old SGCA-null, female and male SGCD-null mice when compared to wild type mice (all n = 5 mice per group). Q, quadriceps; GC, gastrocnemius; TA, tibialis anterior; Dia, diaphragm. * Indicates a significant difference from WT controls. # Indicates a significant difference from female SGCD-null mice Error bars represent standard deviation (±SD).

A hallmark of muscular dystrophy is a high proportion of smaller, regenerative muscle fibers due to cycles of de- and regeneration in patients [18]. To assess fiber size distribution in our animal models, we measured the fiber’s minimal Feret’s diameter in the gastrocnemius, tibialis anterior and diaphragm of LGMD and wild type mice. As expected, the majority of fibers (35–40% depending on the muscle) of SGCA- and SGCD-null mice consisted of smaller fibers with a minimal Feret’s diameter between 5–20 μm (Fig 2a). In contrast, in wild type mice the majority of fibers (30–55% depending on the muscle) consisted of fibers with a minimal Feret’s diameter between 21–40 μm, while only less than 5% of their muscles contained small fibers. Unlike with the hanging tests, the fiber size distribution did not significantly differ between SGCD-null males and females.

#### Increased expression of regeneration markers in LGMD muscles

To detect regenerative fibers, we performed embryonic myosin heavy chain (eMHC) staining in diaphragm muscle from wild type, SGCA- and SGCD-null mice. As expected, diaphragm of SGCA- and SGCD-null mice contained higher levels of eMHC when compared to wild type mice (Fig 2b). To further validate increase in muscle regeneration, we measured gene expression of myogenic markers embryonic myosin heavy chain (*Myh3*), myogenin (*Myog*), signal transducer and activator of transcription 3 (*Stat3*) and myogenic differentiation (*Myod*). As expected, wild type muscles expressed very low levels of these markers, while SGCA- and SGCD-null muscles expressed higher levels (Fig 2c). The increases in *Myog* and *Myh3* levels were found to be significant for LGMD diaphragm muscles. *Stat3* levels were increased to a similar extent in all muscles, but were only significantly elevated in the gastrocnemius muscles of female SGCD-null mice and the tibialis anterior muscles of SGCA-null mice. Similar to *Myog* and *Myh3*, *Myod* was significantly increased in diaphragm muscles of LGMD mice, but also in gastrocnemius muscles of male SGCD-null mice. Unlike with the hanging tests, the myogenic gene expression did not significantly differ between SGCD-null males and females (Fig 2c).

#### Elevated collagen levels in skeletal muscles and heart of LGMD mice

**Fig 3:**
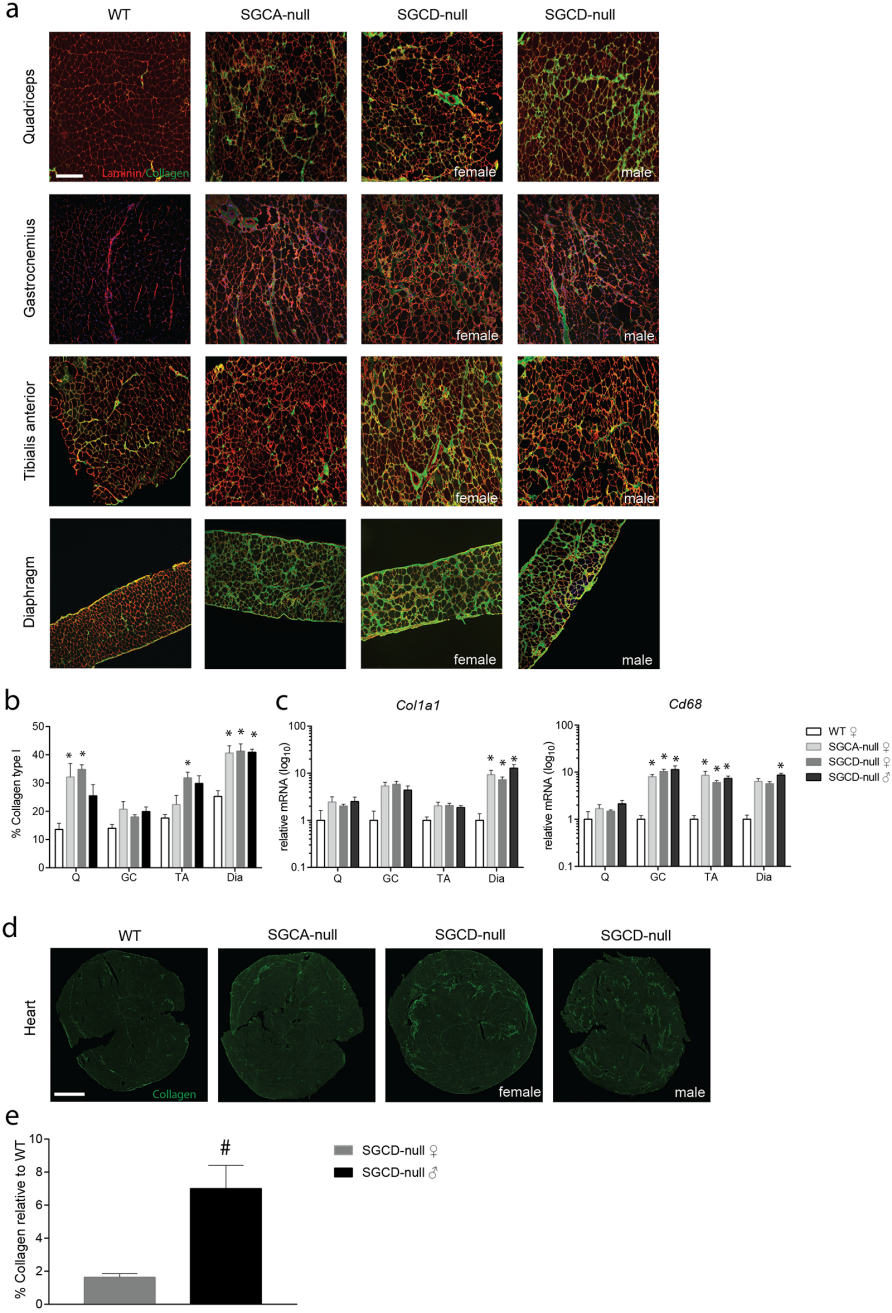
Elevated collagen levels in skeletal muscles in SGCA- and SGCD-null mice and in the heart of SGCD-null mice. **(a)** Immunofluorescent images of skeletal muscles stained with collagen type I (fibrotic marker, green) and laminin (extracellular matrix of muscle fibres, red) Scale bar: 100 μm. **(b)** Quantification of collagen type I positive area in skeletal muscles relative to wild type muscles. A significant increase in the percentage of collagen type I positive area was found in diaphragm and quadriceps muscles of SGCA- and SGCD-null mice, while no difference was found in the gastrocnemius. Tibialis anterior muscles of female SGCD-null mice showed a significant increase in collagen type I positive area compared to wild type. **(c)** Fibrotic and inflammatory gene expression measured by qPCR, normalized to *Gapdh* (n = 5 mice per group) in skeletal muscles of LGMD and wild type mice. A significant increase in *Col1a1* and *Cd68* expression was found in SGCA- and SGCD-null muscles when compared to wild type muscles. **(d)** Immunofluorescence images of the heart stained with collagen type I (fibrotic marker, green). Scale bar: 1000 μm. **(e)** The percentage of collagen type I positive area, measured with Image J was significantly increased in SGCD-null mice compared to wild type mice; males showed a significantly higher increase in collagen than did female SGCD-null mice. Q, quadriceps; GC, gastrocnemius; TA, tibialis anterior; Dia, diaphragm. * Indicates a significant difference from WT controls. # Indicates a significant difference from female SGCD-null mice. Error bars represent ± SD.

Another hallmark of sarcoglycanopathy in patients is fibrosis, the ongoing replacement of the muscle by connective tissue accompanied by chronic inflammation [19]. Fibrosis in SGCA- and SGCD-null mice was examined by looking at collagen type I (*Col1a1*) expression on protein and RNA levels. Overall, muscles of LGMD strains contained extensive fibrotic infiltrates compared to wild type mice, with the diaphragm being most affected (Fig 3a and 3b). Additionally, a significant increase was detected in the quadriceps of female LGMD mice as well as in the tibialis anterior muscles of female SGCD-null mice relative to wild type muscles. We also measured gene expression of *Col1a1* as a marker of fibrosis and cluster of differentiation 68 (*Cd68*) as a marker of inflammation. Interestingly, the gastrocnemius and diaphragm muscles expressed significantly higher *Col1a1* mRNA levels, while the quadriceps and tibialis anterior muscle showed a non-significant elevation of *Col1a1* mRNA levels in both LGMD strains compared to wild type mice (Fig 3c). *Cd68* mRNA levels were significantly increased in LGMD gastrocnemius, tibialis anterior and male SGCD-null diaphragm muscles. In contrast to the hanging tests, no significant differences in fibrosis and inflammation markers were detected between SGCD-null males and females (Fig 3a–3c). In addition to progressive weakness and wasting of the proximal limb muscles, LGMD 2F patients often show cardiac involvement [7]. Therefore, by measuring collagen type I positive areas in heart we studied whether SGCD-null mice develop cardiomyopathy. As expected marked increase in Col1a1 was detected in hearts of SGCD-null mice but it was not the case for SGCA-null and wild type mice (Fig 3d). We quantified Col1a1 positive areas for female and male SGCD-null mice. Notably, in line with the more impaired skeletal muscle function in SGCD-null males, hearts from males contained significantly higher collagen levels than those from SGCD-null females (Fig 3d and 3e).

#### Accumulation of fat infiltrates in muscles of LGMD mice

**Fig 4:**
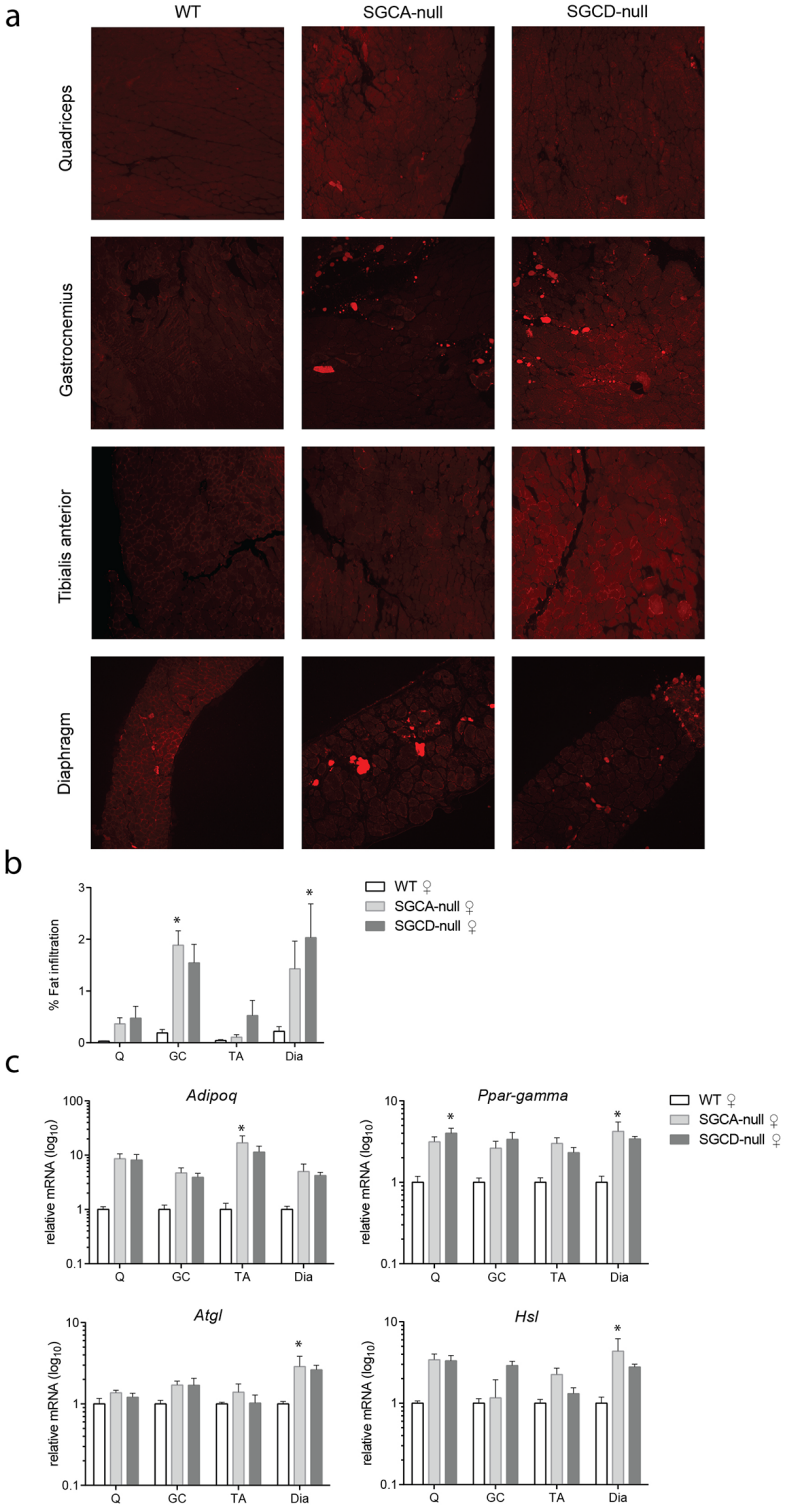
Accumulation of fat infiltrates in LGMD strains. **(a)** Immunofluorescent images of skeletal muscles stained with Nile-red (infiltrated lipids, red) Scale bar: 100 μm **(b)** Quantification of the immunofluorescent areas. A significant increase in Nile red -positive areas (n = 5 mice per group) was found in SGCD-null diaphragm and SGCA-null gastrocnemius muscles when compared to wild type muscles. **(c)** Adipogenic gene expression measured by qPCR, normalized to *Gapdh* (n = 5 mice per group). Upregulation of adipogenic gene expression (*Adipoq*, *Ppar-gamma*, *Atgl*) was found in diaphragm muscle of both LGMD strains and *Hsl* was significantly upregulated in SGCA-null diaphragm muscle compared to wild type. Q, quadriceps; GC, gastrocnemius; TA, tibialis anterior; Dia, diaphragm. * Indicates significant difference from WT controls. Error bars represent ± SD.

When we stained muscle sections with a haematoxylin and eosin staining, we observed round, drop-like, unstained spots in LGMD muscles, but not in healthy muscles. Using Nile-red staining, we identified these spots as infiltrated lipid droplets (Fig 4a) and quantified their abundance in both LGMD strains and wild type mice. Overall, fat infiltrates were more abundant in gastrocnemius and diaphragm than in quadriceps and tibialis anterior muscles of both LGMD strains. The Nile-red positive areas in the gastrocnemius of SGCA-null mice and the diaphragm of SGCD-null mice were significantly larger than in wild type mice (Fig 4b). To confirm our finding, we measured expression of adipogenic genes (Fig 4c). A significant increase of adiponectin (*Adipoq*), which is exclusively expressed in adipose tissue, was observed in tibialis anterior muscles of SGCA-null mice. To distinguish between white and brown adipocytes (key site of heat production), uncoupling protein 1 (*Ucp1*) (expressed only in brown adipocytes) transcript levels were measured. *Ucp1* was not detectable in muscles of wild type and LGMD mice (data not shown) suggesting that observed fat droplets contained mostly white adipocytes. Moreover, we measured expression levels of genes that regulate metabolic processes in adipose tissues. Namely, peroxisome proliferator-activated receptor gamma (*Ppar-gamma*), which is a regulator of adipocyte differentiation, was significantly increased in diaphragm muscle of SGCA-null and quadriceps of SGCD-null relative to wild type mice. Similarly to *Ppar-gamma*, adipose triglyceride lipase (*Atgl* or *Pnpla2*) transcript levels were upregulated in diaphragm muscle of SGCA-null relative to wild type mice. Lastly, hormone-sensitive lipase (*Hsl*), implicated in hydrolyzes of triglycerides, was significantly higher expressed in SGCA-null diaphragm than in wild type muscle. Although not all detected changes reached significance, the expression pattern of the adipogenic genes was consistent in that the gene expression was increased in LGMD mouse models compared to wild type controls.

#### MSTN and TGF-β signalling components contribute to disease pathology in LGMD mice

**Fig 5:**
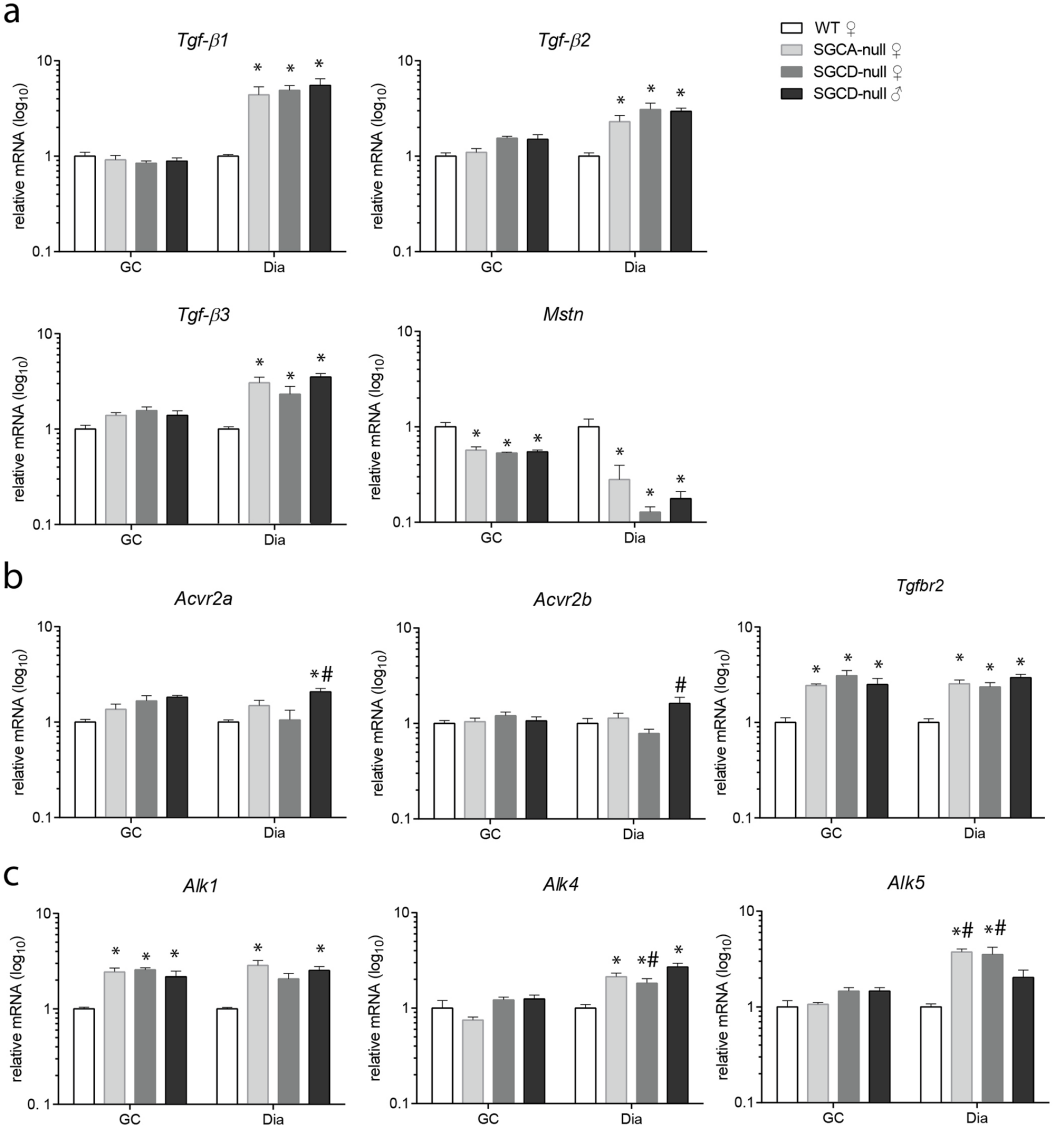
MSTN/TGF-β signalling contributes to LGMD disease pathology. **(a)** Gene expression analysis of three *Tgf-β* isoforms and *Mstn*. *Tgf-β1*, *Tgf-β2* and *Tgf-β3* levels were significantly higher in diaphragm muscles of LGMD compared to wild type, while they did not differ in gastrocnemius muscles. *Mstn* levels were significantly lower in gastrocnemius and diaphragm muscles of LGMD mice. **(b)** Gene expression analysis of type II receptors *Acvr2a*, *Acvr2b* and *Tgfbr2*. *Acvr2a* levels were upregulated in male SGCD-null compared to wild type and female SGCD-null diaphragm muscles. *Acvr2b* levels were significantly higher in diaphragm muscles of SGCD-null males compared to females. *Tgfbr2* expression was significantly increased in gastrocnemius and diaphragm muscles of LGMD compared to wild type mice. **(c)** qPCR analysis of type I receptors: *Alk1*, *Alk4* and *Alk5*. *Alk1* levels were upregulated in LGMD gastrocnemius and SGCA- and male SGCD-null diaphragm muscles compared to wild type muscles. *Alk4* was significantly increased in diaphragm muscles of LGMD mice relative to wild type. In addition, *Alk4* levels were significantly lower in SGCD-null females than males. *Alk5* was significantly higher in diaphragm muscles of SGCA- and female SGCD-null mice relative to wild type and male SGCD-null mice. Data were normalized to *Gapdh* (all n = 5 mice per group). GC, gastrocnemius; Dia, diaphragm. * Indicates a significant difference from WT controls. # Indicates a significant difference from male SGCD-null mice. Error bars represent ± SD.

Elevated activity of the transforming growth factor beta (TGF-β) superfamily contributes to the pathogenesis in various types of neuromuscular disorders by inhibiting muscle repair and promoting an increase in fibrotic tissue formation [20]. We measured gene expressions of TGF-β signalling components and muscle specific growth inhibitor myostatin in the gastrocnemius and diaphragm muscles of SGCA- and SGCD-null mice. Diaphragm muscle of both LGMD strains expressed significantly higher levels of three isoforms of *Tgf-β* (*Tgf-β1*, *Tgf-β2* and *Tgf-β3)* when compared to wild type diaphragm muscle, while no differences were found in the gastrocnemius muscle (Fig 5a). A significant decrease of myostatin (*Mstn*) was detected in both muscles of SGCA- and SGCD-null mice when compared to wild type mice, but this decrease was larger in diaphragm muscles. No significant differences were detected between male and female SGCD-null mice. We also measured gene expression levels of myostatin and TGF-β type II and type I receptors. Gender-specific differences in activin A receptor type 2A and 2B (*Acvr2a* and *Acvr2b*) levels were detected in SGCD-null diaphragm muscle, where males expressed significantly higher mRNA levels than females (Fig 5b). Additionally, *Acvr2a* was significantly upregulated in male SGCD-null relative to wild type diaphragm muscle. A significant upregulation of transforming growth factor beta receptor 2 (*Tgfbr2*) was detected in both muscles of SGCA- and SGCD-null mice when compared to wild type mice. *Alk1* was upregulated in the gastrocnemius and diaphragm muscle of LGMD mice (Fig 5c). *Alk4*, which was previously described as a specific myostatin type I receptor in muscles [21, 22] was significantly increased in LGMD diaphragm muscle. Similar to *Acvr2a* and *Acvr2b* results, SGCD-null males expressed significantly higher *Alk4* levels than did females. *Alk5* levels were significantly higher in diaphragm of SGCA- and female SGCD-null mice compared to wild type and male SGCD-null mice.

To summarize, skeletal muscles of both LGMD strains contained a higher number of small regenerative fibers than did wild type mice, which was further confirmed by increased expression levels of myogenic genes. In addition, fibrotic and fat infiltrates had accumulated in LGMD muscles. Male SGCD-null mice showed more severe cardiac fibrosis than did female mice. Moreover, TGF- β signalling components were increased, while *Mstn* levels were downregulated in LGMD muscles. All these dystrophic hallmarks were most pronounced in diaphragm muscle.

#### Functional test regime does not interfere with the muscle pathology

**Fig 6:**
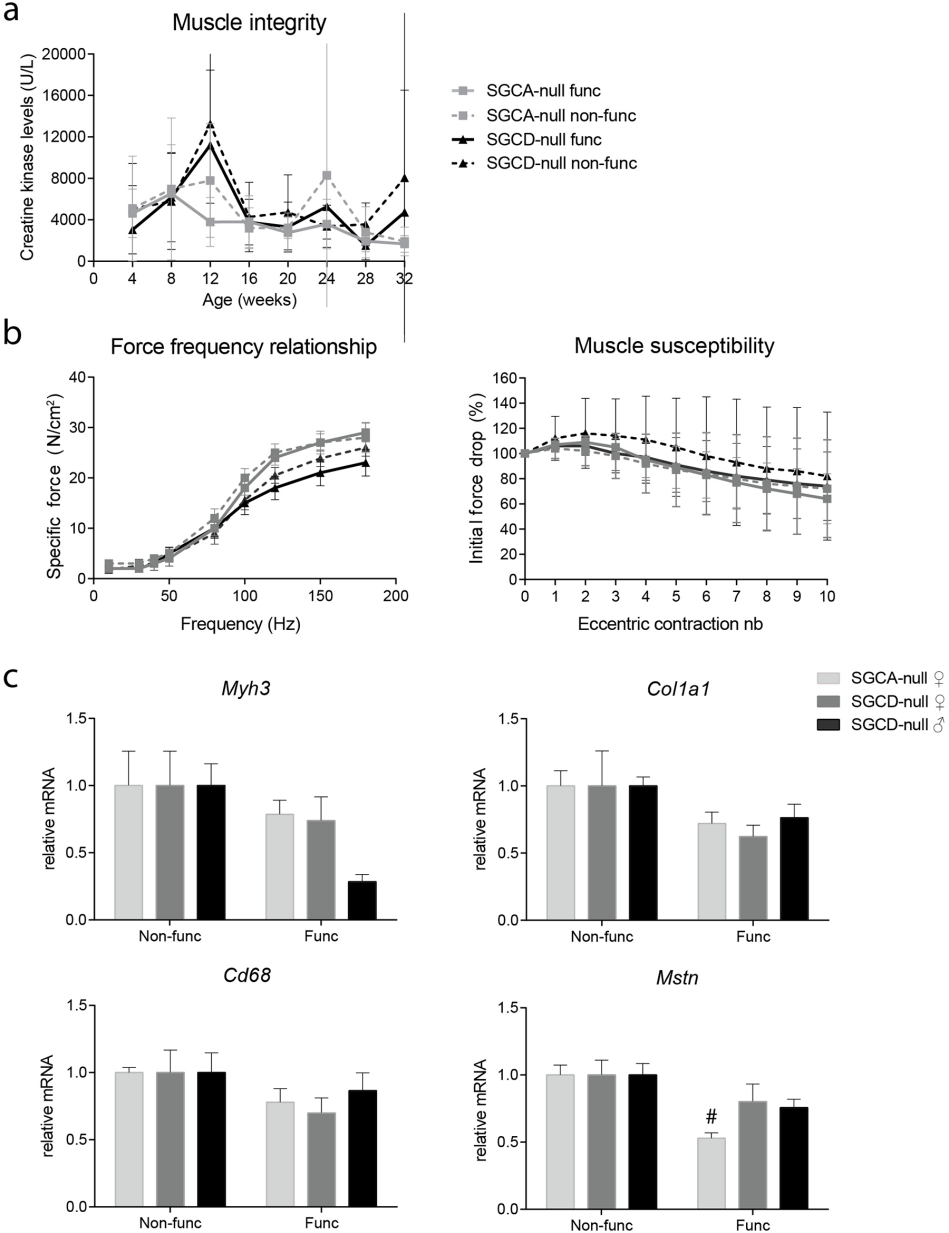
Functional test regime did not interfere with muscle pathology in both LGMD strains. **(a)** CK levels were similar in functionally challenged and sedentary mice. **(b)** Tibialis anterior physiology of the functional challenged and sedentary groups. The functional test regime did not have an effect on muscle physiology **(c)** Gene expression analysis of pathogenic markers in gastrocnemius muscle of functionally challenged and sedentary LGMD mice. *Myh3*, *Col1a1* and *Cd68* mRNA levels did not differ between functionally challenged and sedentary groups. *Mstn* levels were lower in functionally challenged compared to sedentary SGCA-null mice. # Indicates a significant difference from non-functional SGCA-null mice. Error bars represent in a, b ± SEM and in c ± SD.

To assess whether the functional test regime interfered with disease pathology in both LGMD strains, similar sized sedentary groups were included. We measured muscle integrity, muscle physiology and expression of genes involved in LGMD muscle pathogenesis in these mice. The functional test regime did not interfere with muscle integrity in both LGMD strains, as CK levels were comparable between functional test regime and sedentary groups (Fig 6a). Moreover, the functional test regime also did not affect physiology of tibialis anterior muscles (Fig 6b). Lastly, gene expression levels did not differ between the groups, except for *Mstn* levels, which were significantly decreased in functionally challenged compared to sedentary SGCA-null mice (Fig 6c).

## Discussion

Currently, there is no therapy available for LGMD types 2D and 2F and clinical management is similar to that described in care guidelines for DMD [19, 23]. A number of animal models of α- and δ-sarcoglycanopathies have been developed with the aim to understand the mechanism of disease pathogenesis and to identify therapeutic targets. Genetically engineered mice deficient for either α-sarcoglycan (SGCA-null) or δ-sarcoglycan (SGCD-null) are suitable models for pre-clinical research because they recapitulate many features of the human disease [7, 8]. However, the crucial elements for evaluation of drug benefits are still lacking, including comprehensive natural history data and clearly defined outcome measures. This hinders pre-clinical research in LGMD mouse models. To address this shortcoming, we collected natural history data and defined outcome measures for α- and δ–sarcoglycan deficient mice in the present study.

Mice underwent longitudinal, non-invasive muscle function assessments in a reliable and reproducible manner, as previously described for *mdx* mice [12], allowing us to monitor disease progression and treatment effects over time [24]. We found that the two and four limb hanging tests were sensitive and reliable outcome measures, as we detected marked differences in the results between wild type and both LGMD strains with high statistical significance. Namely, we observed a significant muscle function impairment already at four weeks of age in both LGMD strains, confirming previous findings that sarcoglycan deficient mice have an early disease onset [25]. Moreover, these differences increased over time. Although, both female and male SGCD-null mice fully manifest the disease phenotype, we found that there were gender differences that influenced muscle function assessments of SGCD-null mice. Therefore, we advise to use males for pre-clinical studies, or alternatively large equally sized groups for each gender and treatment arm to minimize variation.

Results of the four limb grip strength test were significantly different between wild type and SGCD-null mice, while this test was less effective for SGCA-null mice. We suggest using four limb grip strength as an outcome measure, but only for SGCD-null mice. In contrast to both hanging tests, the four limb grip strength test was ineffective in detecting a decline in muscle function over time, thus this test is not useful for measuring disease progression.

We also identified functional assessments that are less useful. Rotarod testing analysis did not reveal any significant differences in SGCA- and SGCD-null mice relative to wild type mice. For *mdx* mice, limitations and possible optimizations of rotarod testing have been previously discussed [24]. There and here, high variability between individual mice did not permit comparison between groups or across studies. We therefore propose to exclude rotarod testing as an outcome measure for pre-clinical trials in LGMD mice. The beam walk test has been used to assess motor skills in mice with cortical impact lesions [26] and in Huntington disease mouse models [27, 28]. To our knowledge this test has never been performed in LGMD strains. In this study, we observed an increase in traverse time and hind paw slips in LGMD relative to wild type mice. However, these differences did not reach significance. We think that the beam walk test is a reliable and sensitive measure for balance and coordination, but more suitable in mouse models for nervous system disorders than muscular dystrophies.

CK levels are a widely used biomarker for fiber damage. Similar to LGMD patients, the sarcoglycan-deficient mice showed elevated CK levels. However, CK is a very global measure of muscle damage and tends to be variable based on e.g. animal activity [24]. Due to these limitations, it is difficult to correlate the CK values to disease progression. Although we were able to detect worsening of muscle function with age in LGMD mice, CK levels were highly elevated but remained unchanged over time.

Respiratory dysfunction is observed in LGMD patients at an advanced stage of the disease and is associated with reduced life expectancy [29]. Impairment in respiratory function was also clearly detected in SGCA- and SGCD-null mice. Contrary to the human situation, we observed a deficit already at an earlier stage.

We assessed specific and isometric force contraction in tibialis anterior muscles. Confirming our findings from non-invasive functional tests, these analyses showed a marked impairment in muscle function and higher susceptibility to muscle damage in LGMD compared to wild type mice. Such functional assessments are valuable tools for validation of muscle function and treatment benefits.

We here show that active muscle regeneration persists in 34-week-old mice and thereby may counteract progressive muscle loss. In contrast, muscle regeneration in older LGMD 2D and 2F patients is impaired, leading to severe myopathy and premature death [6, 29, 30]. Furthermore, we examined levels of fibrosis in LGMD mice using a collagen type I staining. We detected marked differences between LGMD and wild type mice and between different muscles. However, the method used here overestimates the amount of collagen in dystrophic muscles, since collagen present in the endomysium (both in wild type and dystrophic muscles) is included in the calculation. Moreover, we found that muscles of both LGMD strains contained fat droplets, with the highest level in the diaphragm. Our finding concurs with the previous electron microscopy study that found fat droplets in the diaphragm from SGCA-null mice [31]. We think that it is a specific hallmark for sarcoglycan mutants, since to our knowledge it has never been described in *mdx* mice and other muscular dystrophy mouse models [25]. Histological evaluation of fat droplets differed from the gene expression analyses of fat-related genes, where fat droplets were mainly detected in gastrocnemius and diaphragm muscles, while *Ppar-gamma* was upregulated in quadriceps and diaphragm muscle and *Adipoq* was increased in tibialis anterior muscle. This discrepancy between histological and gene expression data can be explained by the fact that we could have lost fat droplets during the sectioning procedure, as muscle was sectioned at -20°C while fat is usually cut at lower temperature.

Currently, the muscle type used for histological examinations varies between laboratories as there is no agreement about which muscle type best mimics the histological phenotype observed in patients with LGMD. In our study, we compared quadriceps, gastrocnemius, tibialis anterior and diaphragm muscles of SGCA- and SGCD-null mice. All muscle dystrophy hallmarks examined were more pronounced in diaphragm muscles. Interestingly, this is in line with a number of other muscular dystrophy mouse models [25]. The diaphragm is thought to be the most frequently used muscle, which would explain the more severe contraction induced damage. When we compared between limb muscles, the quadriceps and gastrocnemius were affected to a similar extent, whereas the tibialis anterior muscle showed a milder phenotype. Therefore, we conclude that there is a specific pattern of muscle type involvement in LGMD mice which needs to be taken into consideration in pre-clinical test designs. However, the exact cause of this specific pattern remains unclear It has been shown that muscle fiber type composition can influence disease progression. In DMD patients and *mdx* mice, the fast-glycolytic fibers are more susceptible to contraction induced damage and the first to degenerate, whereas the slow-oxidative fibers are more protective against damage [32, 33]. This means that fast muscles (e.g. extensor digitorum longus) are earlier and more severely affected by the disease than slow fiber-rich muscles (e.g. soleus) [34]. However, our studied limb muscles contain comparable mixture of fiber types, mostly fast type IIb fibers. Thus, this cannot explain the observed difference in disease severity between these muscles. Interestingly, the mild phenotype of tibialis anterior muscle was also observed in *mdx* mice when compared to other limb muscles [11]. As mentioned previously for diaphragm, frequent muscle usage contributes to more severe muscle damage. However, differences in usage of limb muscles and other factors contributing to distinguished muscle type involvement remain to be further investigated.

Several members of the TGF- β and myostatin signalling pathways have been implicated as therapeutic targets for a large group of neuromuscular disorders, including LGMD [20]. Moreover, activity of TGF- β signalling modifies the severity of disease pathology [35]. We conclude that the diaphragm—being the more severely affected muscle and expressing higher levels of TGF- β isoforms—provides a suitable model for studying how the TGF- β signalling mediates the adverse effects on muscle pathology in LGMD mouse models. In contrast to TGF-β, myostatin, a muscle-specific secreted peptide serving as muscle growth inhibitor [36], was decreased in LGMD skeletal muscles. Furthermore, decrease in myostatin was associated with more severe disease phenotype. Therefore, it is debatable whether myostatin inhibition will be beneficial at an advanced stage of disease. In a previous study, early myostatin inhibition was beneficial but this treatment had no therapeutic effect in older LGMD mice [37].

Since most parameters assessed remained unchanged between sedentary and functional challenged groups, we concluded that the functional test regime did not interfere with the disease pathology. Notably, *Mstn* transcript levels were decreased in functionally challenged SGCA-null mice. On one hand, we think that *Mstn* levels may be modulated by the functional test regime, since myostatin is profoundly involved in whole body metabolism and training results in a decreased myostatin expression [38]. On the other hand, it has been demonstrated that *Mstn* expression was not affected by chronic treadmill exercise in *mdx* mice, which is much more detrimental to the mice than the functional test regime that was used in the present study [39]. We postulate that the effect on *Mstn* expression might be genotype-specific, as it was only observed in SGCA-null mice. However, this needs to be further validated.

Cardiac involvement has been reported in 17% to 50% of patients with sarcoglycanopathies [40]. SGCD-null mice, likewise develop cardiomyopathy with focal areas of fibrosis due to abnormalities in the coronary vasculature (18). While SGCA-null mice display progressive muscular dystrophy, but no cardiac abnormalities [9]. We indeed detected cardiac fibrosis in SGCD-null but not in SGCA-null mice. The mechanisms underlying differences in pathogenesis of cardiomyopathy between SGCA- and SGCD-null mice were previously reported [8, 41, 42]. The main difference is that δ-sarcoglycan is strongly expressed in the smooth muscle cells, in contrast to the α-sarcoglycan. Thus, the deficiency for δ-sarcoglycan in SGCD-null mice results in loss of vascular smooth muscle complex leading to the disruption of the coronary vasculature and the development of myocardial necrosis [8]. Interesting, we found that cardiac fibrosis was more pronounced in males than in females, which positively correlates with the performances in the limb hanging tests. We therefore would advise the use of SGCD-null males in pre-clinical studies assessing therapeutic effects of cardioprotective drugs for LGMD 2F.

## Conclusion

Our study offers a comprehensive natural history dataset, based on which we made a list of recommendations to be considered in conducting pre-clinical experiments in SGCA- and SGCD-null mice.

1. Age of treatment: We recommend to start treatment in both young mice (four weeks of age) and in older mice (16 weeks of age). Since, muscle function is impaired at 4 weeks of age and decline is detectable between 14–16 weeks of age.
2. Sample size: Sufficient numbers of mice must be included to detect statistically significant differences. Based on our statistical analyses, we concluded that the exact numbers depend on the variability of each outcome measure. Functional *in vivo* tests show higher variability and should include at least six mice per group. Terminal outcomes such as histological and gene expression analyses are less variable and require a minimum of five mice per group.
3. Gender selection: We advise to use SGCD-null males for pre-clinical trials, as they provide a larger therapeutic window (see Results from the hanging tests and measured levels of cardiac fibrosis). Although SGCA-null mice did not show any significant differences between genders, we advise to use single gender (preferably males) for pre-clinical therapeutic trials to minimize variation, or use large sample sizes of both genders.
4. Muscle selection: As different muscle types are not equally affected, we recommend to include several different muscles in the treatment evaluation, e.g. diaphragm (as the most severely affected muscle) and at least one of the limb muscles (quadriceps or gastrocnemius).
5. Outcome measures: Functional *in vivo* tests such as hanging tests and whole-body plethysmography are informative for both LGMD strains, whereas grip strength tests are less applicable to SGCA-null mice. We do not recommend the use of rotarod or beam walk tests. Defined terminal outcome measures include specific and isometric force contraction assessments in tibialis anterior muscles, histological analyses like fiber size distribution, fibrosis and fat droplets and expression analyses of genes involved in muscle regeneration, fibrosis, inflammation and adipogenesis.

## Supporting information

S1 Fig Experimental timeline. Grey squares indicate time points at which functional test regime was performed. Black stars denote time points at which blood samples were collected for CK measurements. Respiratory function was conducted in mice at 15 and 34 weeks of age indicated by black circles. Muscle physiology and terminal analyses were performed at 34 weeks of age indicated by black square.
(TIF)

S2 Fig Functional test performances of SGCA- and SGCD-null mice. **(a)** Normalized four limb grip strength was significantly decreased in SGCD-null mice than in wild type and SGCA-null mice **(b)** Maximum running time on the rotarod was shorter in both LGMD strains when compared to wild type mice. SGCA-null mice performed worse than SGCD-null mice in the rotarod test. **(c)** Maximum hanging time with two limbs was significantly shorter in SGCA- and SGCD-null mice when compared to wild type mice. SGCD-null mice performed better than SGCA-null mice did. **(d)** Maximum hanging time with four limbs was significantly decreased in SGCA- and SGCD-null mice when compared to wild type mice. SGCD-null mice outperformed SGCA-null mice. **(e)** Traverse latency (seconds) was higher and increased with age in SGCA- and SGCD-null compared to wild type mice. **(f)** The number of hind limb slips was higher and increased with age in both LGMD strains compared to wild type mice. **(g)** Body weights recorded over time for wild type, SGCA- and SGCD-null mice. * Indicates a significant difference from wild type (WT) controls. # Indicates a significant difference from SGCA-null mice. Error bars represent standard error of the mean.
(TIF)

S1 Checklist This file contains ARRIVE (Animal Research: Reporting of *In Vivo* Experiments) guidelines checklist that is required for reporting of research using animals. (DOCX)

## Abbreviations

CK: Creatine kinase
DGC: Dystrophin-glycoprotein complex
LGMD: Limb-girdle muscular dystrophy
LGMD2D: Limb-girdle muscular dystrophy type 2D
LGMD2F: Limb-girdle muscular dystrophy type 2F
Sgca: α-sarcoglycan
SGCA-null: α-sarcoglycan deficient mice
Sgcd: δ-sarcoglycan
SGCD-null: δ-sarcoglycan deficient mice
