# Supplementary figures and images for: Natural disease history of mouse models for limb girdle muscular dystrophy types 2D and 2F
Source: PLoS One. 2017 Aug 10;12(8):e0182704. doi: 10.1371/journal.pone.0182704 (PMC5552258; doi:10.1371/journal.pone.0182704)

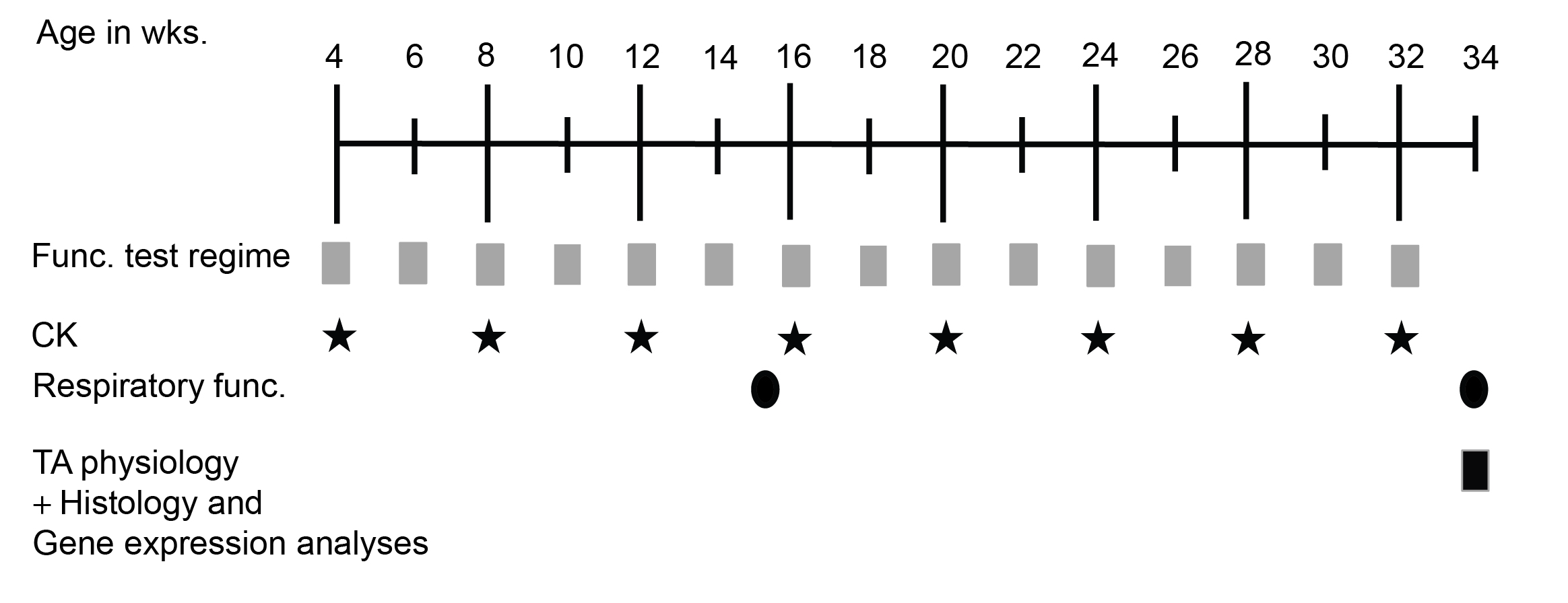

Supplement: S1 Fig — Grey squares indicate time points at which functional test regime was performed. Black stars denote time points at which blood samples were collected for CK measurements. Respiratory function was conducted in mice at 15 and 34 weeks of age indicated by black circles. Muscle physiology and terminal analyses were performed at 34 weeks of age indicated by black square. (TIF) [file pone.0182704.s001.tif]

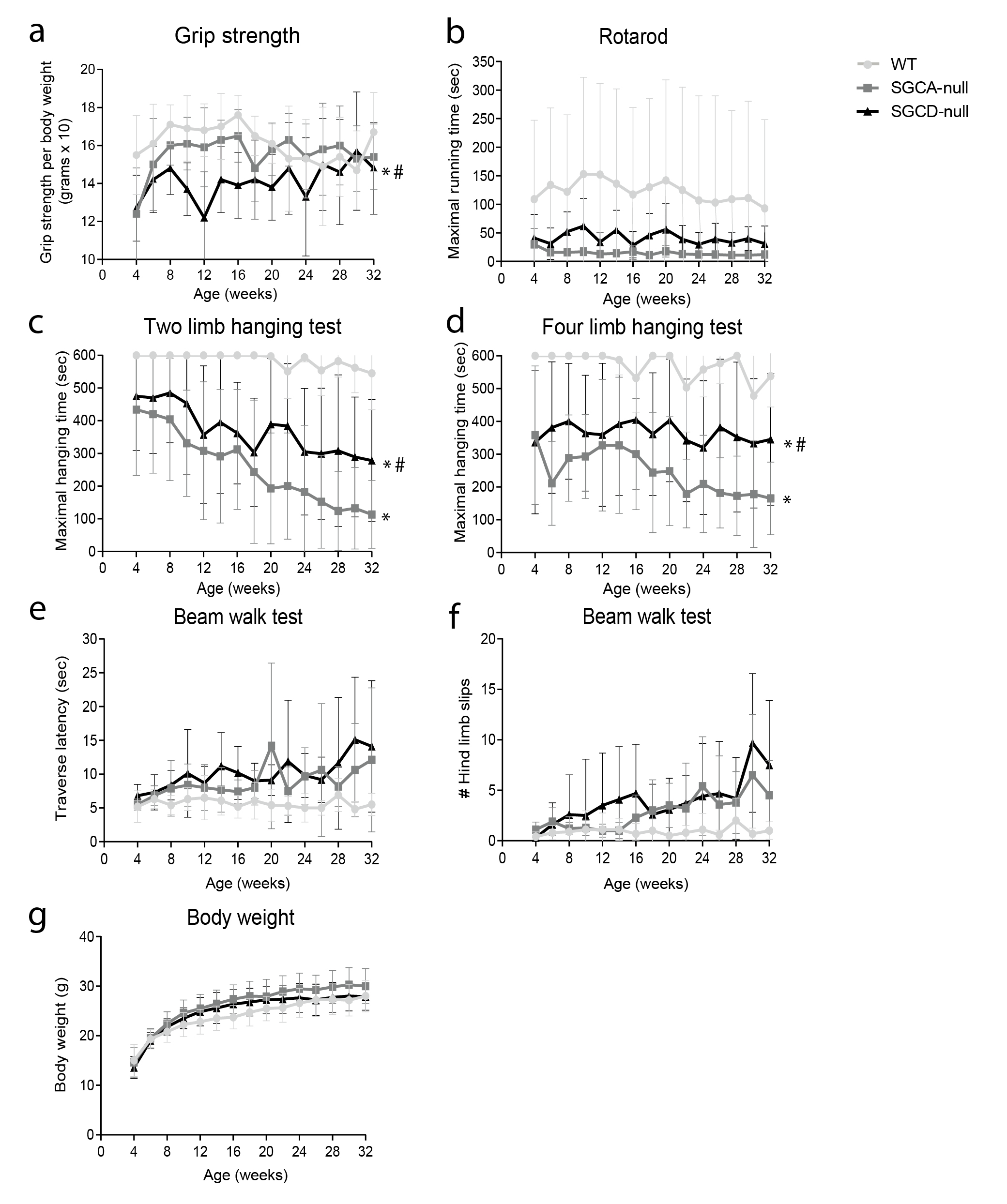

Supplement: S2 Fig — (a) Normalized four limb grip strength was significantly decreased in SGCD-null mice than in wild type and SGCA-null mice (b) Maximum running time on the rotarod was shorter in both LGMD strains when compared to wild type mice. SGCA-null mice performed worse than SGCD-null mice in the rotarod test. (c) Maximum hanging time with two limbs was significantly shorter in SGCA- and SGCD-null mice when compared to wild type mice. SGCD-null mice performed better than SGCA-null mice did. (d) Maximum hanging time with four limbs was significantly decreased in SGCA- and SGCD-null mice when compared to wild type mice. SGCD-null mice outperformed SGCA-null mice. (e) Traverse latency (seconds) was higher and increased with age in SGCA- and SGCD-null compared to wild type mice. (f) The number of hind limb slips was higher and increased with age in both LGMD strains compared to wild type mice. (g) Body weights recorded over time for wild type, SGCA- and SGCD-null mice. * Indicates a significant difference from wild type (WT) controls. # Indicates a significant difference from SGCA-null mice. Error bars represent standard error of the mean. (TIF) [file pone.0182704.s002.tif]
