# Supplementary material for: Natural disease history of mouse models for limb girdle muscular dystrophy types 2D and 2F
Source: PLoS One. 2017 Aug 10;12(8):e0182704. doi: 10.1371/journal.pone.0182704 (PMC5552258; doi:10.1371/journal.pone.0182704)

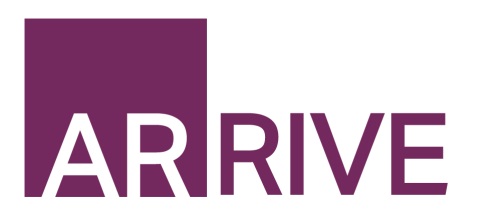


The ARRIVE Guidelines Checklist

Animal Research: Reporting In Vivo Experiments

S. Pasteuning-Vuhman^1^, K. Putker^1^, C.L. Tanganyika-de Winter^1^, J.W. Boertje-van der Meulen^1^, L. van Vliet^1^, M. Overzier^1^, J.J. Plomp^2^, A. Aartsma-Rus^1^, M. van Putten^1^

^1^ Department of Human Genetics Leiden University Medical Centre, Leiden, The Netherlands ^2^ Department of Neurology Leiden University Medical Centre, Leiden, The Netherlands.

|  | | ITEM | RECOMMENDATION | Section/ Paragraph |
| --- | --- | --- | --- | --- |
| 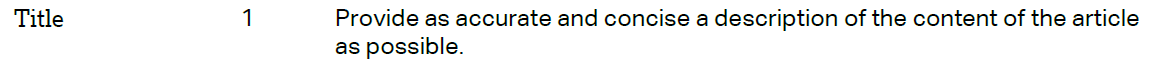 | | | Title |  |
| 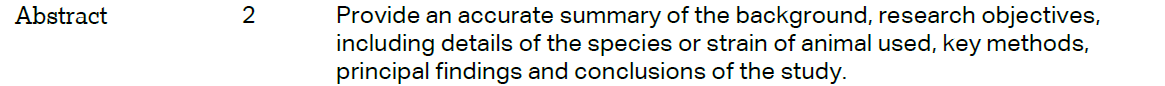 | | | Abstract |  |
| INTRODUCTION | | |  |  |
| 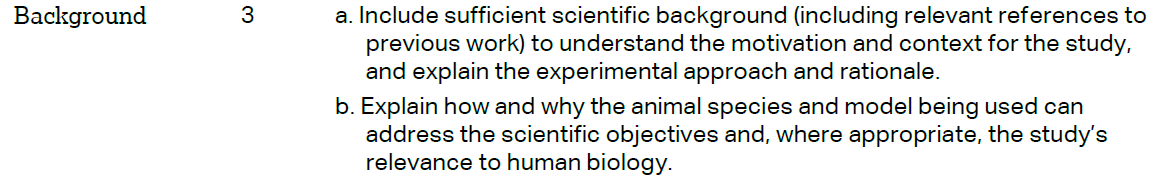 | | | Paragraphs 1-2  Paragraphs  2-3 |  |
| 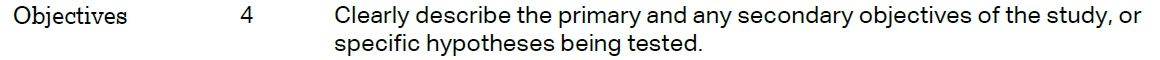 | | | Paragraph 3 |  |
| METHODS | | |  |  |
| 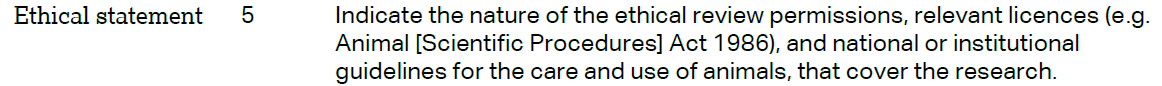 | | | Paragraph 1 |  |
| 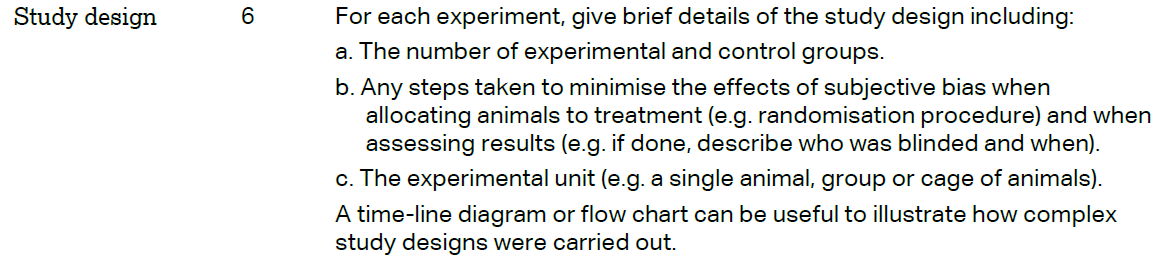 | | | Paragraph 1  Paragraphs  1-2  Paragraph 2  S1 Figure |  |
| 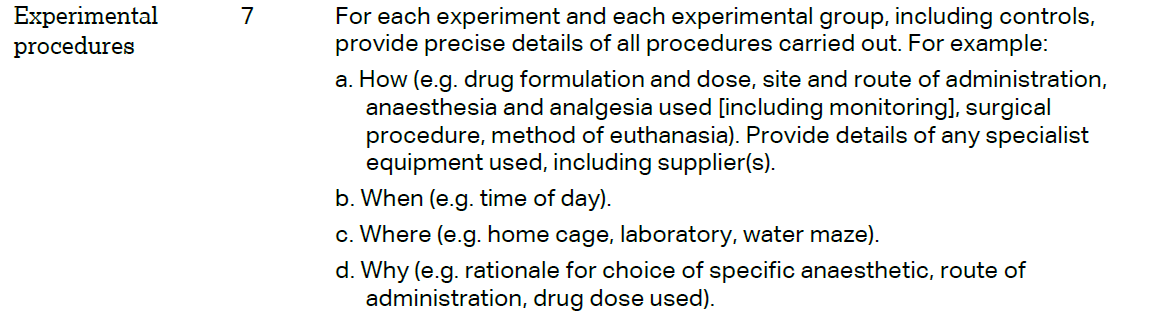 | | | Paragraphs  2-10 |  |
| 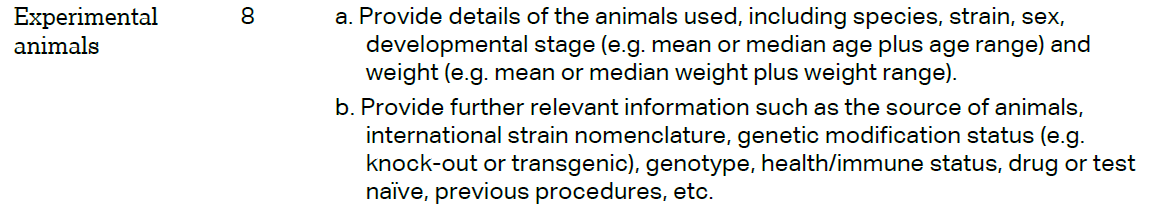 | | | Paragraphs  1-2  Paragraph 1 |  |

The ARRIVE guidelines. Originally published in *PLoS Biology*, June 2010^1^

| 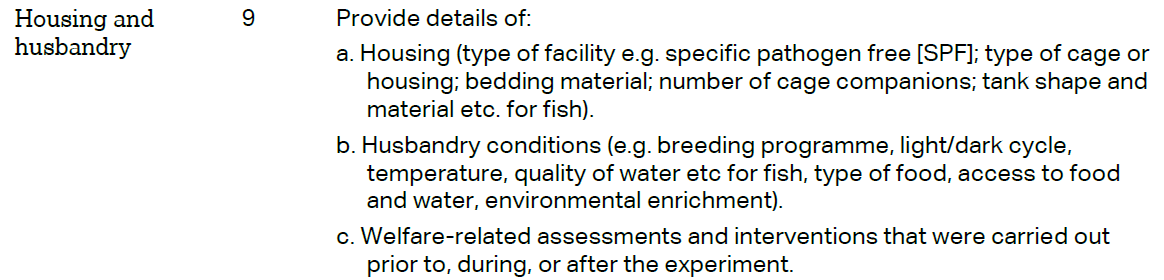 | Paragraphs 1-2  Paragraphs 1-2  Paragraphs 1 and 10 | |
| --- | --- | --- |
| 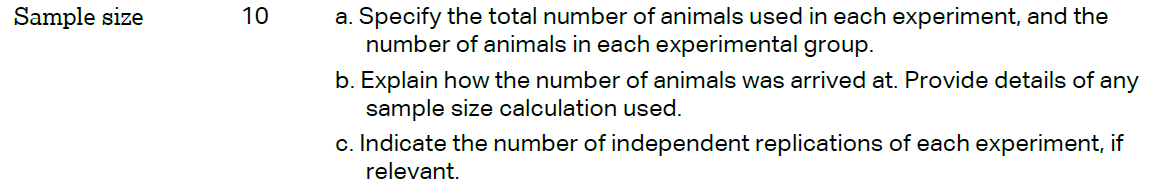 | Paragraph 2 | |
| 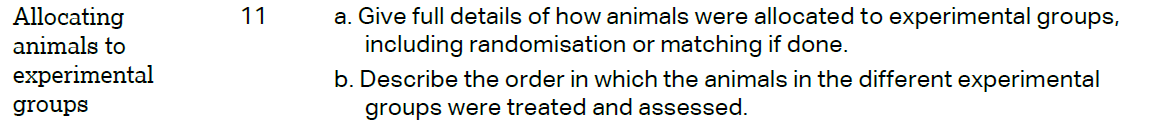 | Paragraphs  1-2 | |
| 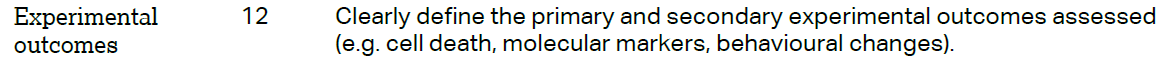 | Paragraphs 3-12 | |
| 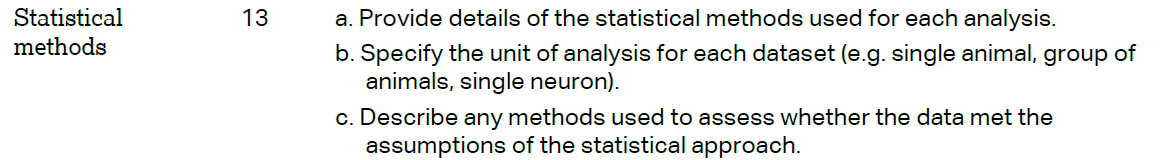 | Paragraph 13 | |
| RESULTS |  | |
| 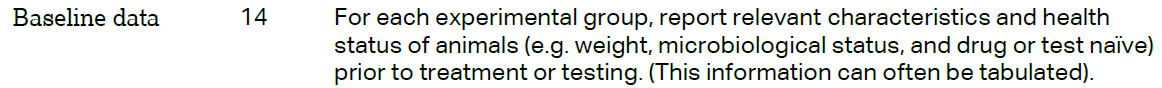 | Figure S2d | |
| 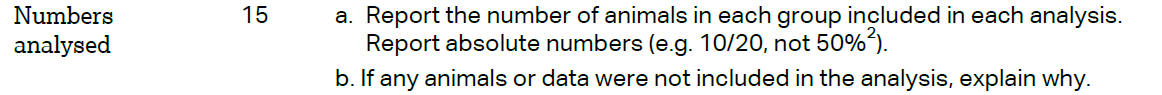 | Paragraph 1 | |
| 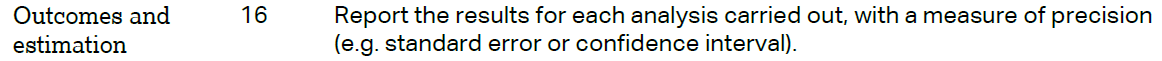 | Figures 1-6  Figure S2 | |
| 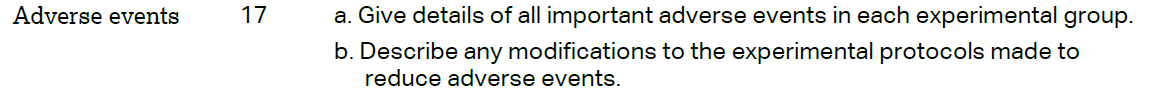 | No adverse events were observed | |
| DISCUSSION |  | |
| 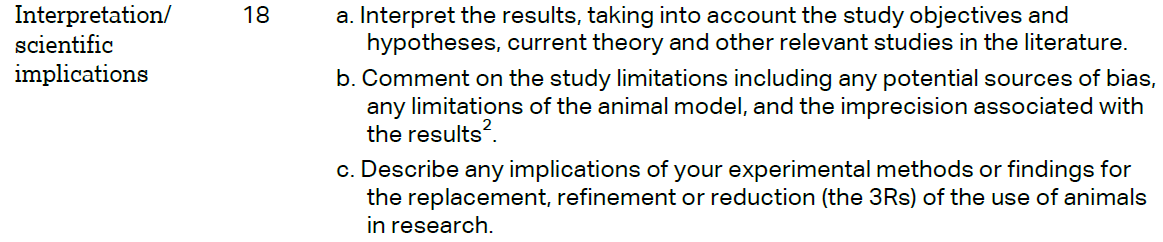 | Discussion  Paragraphs 3,4 and 7  Conclusion | |
| 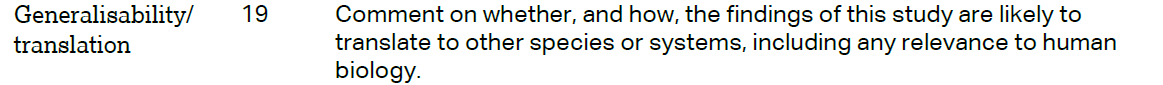 | Conclusion  (These data useful for future preclinical studies) | |
| 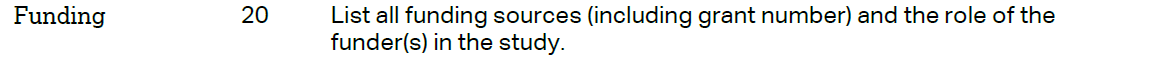 | | This work was supported by grants from ZonMw (Project 113302001),NeurOmics (FP7, Agreement 2012-305121) and AFM (grant number 20251). |


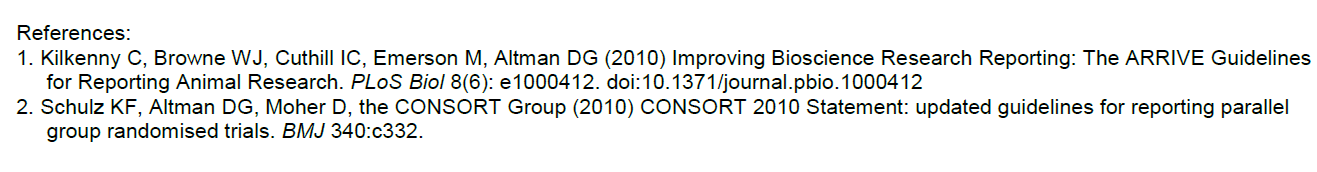

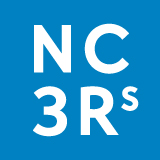

Supplement: S1 Checklist — (DOCX) [file pone.0182704.s003.docx]
